# Rapid spread of Chikungunya virus infection in Orissa: India

**Published:** 2011-03

**Authors:** B. Dwibedi, J. Sabat, N. Mahapatra, S.K. Kar, A.S. Kerketta, R.K. Hazra, S.K. Parida, N.S. Marai, M.K. Beuria

**Affiliations:** *Regional Medical Research Centre (ICMR), Bhubaneswar, India*

**Keywords:** *Aedes*, CHIK, epidemic, fever, Orissa

## Abstract

**Background & objectives:**

A large number of cases of undiagnosed fever and joint pain were reported from different parts of the State of Orissa since February 2006. Epidemiological and laboratory investigation were carried out to confirm the cause of emerging illness, which was provisionally suspected as Chikungunya (CHIK) fever.

**Methods:**

Upon getting the reports of suspected CHIK like illness in different parts of the State, epidemic investigations were carried out in the outbreak affected villages. Case history was recorded, clinical examination undertaken and blood samples collected for seroconfirmation for CHIK IgM antibody using ELISA based kit. Simultaneously vector survey was also carried out.

**Results:**

With no previous record of CHIK infection in the State, the first outbreak was confirmed during February 2006. Subsequently, the infection spread to 13 of 30 districts in different episodes covering 79 villages till November 2007. Attack rate was 9-43 per cent in the different outbreaks with average seropositivity of 24 per cent to CHIK specific IgM. Morbidity was high though no deaths were recorded. *Aedes aegypti* and *Ae. albopictus* were identified as the possible vectors for transmission.

**Interpretation & conclusions:**

The report confirmed emergence of CHIK infection in the State of Orissa, India, and its spread to a larger geographic zone in a short period which warrants public health measures to control further spread.

Chikungunya (CHIK) virus is an Arbo virus of the genus Alphavirus and family *Togaviridae*^1^,^2^. Infection due to CHIK virus results in a debilitating but non-fatal illness in form of epidemics in favoured areas with abundance of the vector species *i.e., Aedes aegypti* and *Ae. albopictus*. It was suspected to be originated from Africa and was isolated for the first time from the blood of a febrile patient during the Tanzanian outbreak in 1952^3^. Since that time it has been reported to be the cause of many epidemics in Western, Central and Southern Africa and many parts of Asia^4^.

In India, major epidemics of CHIK fever were reported during 1963-1973 affecting States like West Bengal, Maharastra, Tamil Nadu, Andhra Pradesh, Madhya Pradesh and Puducherry. The infection re-emerged as epidemics after a silence of 32 years *i.e*., in 2005 affecting many States in the country^5^.

With no previous record of CHIK infection from Orissa (India), sudden onset of cases with fever and joint pain appeared in large numbers in different parts of the State in 2006-2007. Investigation was carried out to identify the cause of the epidemics, which was suspected as CHIK infection. This report briefly presents about the confirmed CHIK outbreaks from different parts of the State, beginning with the first report from February 2006 until November 2007.

## Material & Methods

*Study area:* The state of Orissa is located on the East coast of India in between 17° 48’ and 22° 34’ North latitude and 81° 24’ and 87° 29’ East longitude. The investigation was carried out in the affected parts situated in both the coastal planes and hilly region of the State. The study covered 33 affected blocks from 13 of 33 districts namely Sundergarh, Gajapati, Bhadrak, Ganjam, Jajpur, Kendrapada, Nayagarh, Khurda, Balasore, Puri, Cuttack, Keonjhar and Jagatsinghpur. This investigation was undertaken in different episodes as per the report of CHIK suspected cases detected during the period from February 2006 to November 2007.

*Epidemiological investigation:* Field investigation was carried out by the team from RMRC comprising of clinicians, epidemiologist, entomologists, and laboratory and census personnel. Necessary assistance was sought from State Health Epidemic Response Team while investigating the outbreaks for identification of villages, case enumeration and in some areas for sample collection and follow up of the individual cases. Clinical and epidemiological examination was done by house-to-house visit in the affected villages. The individuals with symptoms of suspected CHIK virus infection were enlisted and examined. An individual presenting with sudden onset of fever and/or joint pain with or without associated symptoms like myalgia, rash and swelling of joints, was considered as a clinical case for CHIK fever^6^. Detailed history and observations were recorded including date of onset of illness, migration and probable exposure, family affection, course of disease, symptoms and signs, recovery of illness and treatment received. Symptomatic treatment was provided to the affected people by the team doctors. Blood sample (4-5 ml) was collected from the willing individuals after obtaining informed written consent for laboratory confirmation. Thick blood smears were collected for examination of malaria parasite. The study was approved by the Institutional Ethics Committees.

*Entomological investigation:* Entomological survey was conducted in the villages by household visit for presence of vector mosquitoes *Aedes* species known to cause CHIK virus transmission for both adult and larvae. Collection of resting adult was carried out from different locations inside of the house and cow sheds, using sucking tube and mechanical aspirator^7^. Adults were identified using keys of Barraud^8^. For collection of larvae, all containers with water were searched inside and outside the houses using dip method or by a Pasteur pipette. Collected larvae were reared to adults and identified. The per man hour density (PMHD) for each species was calculated as number of mosquitoes collected by a man in one hour. Larval collection data were used to calculate house, container and Breteau indices using standard formulae^6^.

*Laboratory diagnosis:* Serum samples were tested for CHIK and dengue IgM antibody using IgM antibody capture ELISA kit produced by National Institute of Virology (NIV), Pune, India^4^. Antigens from African strain of CHIK virus and dengue serotype 2 were used for the respective diagnostic kits. The tests were carried out following the manufacturer instruction. The sample was considered positive for IgM antibody when sample optical density (OD)/negative OD was > 2.1. Both positive and negative controls were used to validate the test. Thick blood smears were examined for presence of malaria parasite in the nearest primary health centre and a part cross-checked at RMRC laboratory.

*Statistical analysis:* Chi-square test was used to test for association.

## Results

*Outbreak period, area and population affected:* Outbreaks of CHIK were recorded from 13 of the 30 districts of Orissa covering 33 revenue blocks and 78 villages. It was reported for the first time from Panposh town of Sundergarh District. Subsequently it was observed from four more districts during 2006 and 11 districts during 2007 (Table I).The epidemics were reported from both small and big villages/ towns with population ranging from 200>5000. Attack rate ranged from 0.4 to 50.76 per cent in the different villages. The outbreaks were observed to continue for 6 days to 2 months in different areas/episodes. A total of 10867 individuals were recorded to be affected out of 77,866 population at risk in the affected areas. Appearance of CHIK cases in the State, is shown in a weekly epidemic plot in the Fig. It indicated that except the first major outbreak which occurred during spring (February & March), majority of cases were reported in the months of September to November in both the calendar years *i.e*., 2006 and 2007, which coincided with late or post monsoon period. Population from all ages and both sexes were shown to be affected from CHIK illness (Table II). Comparatively more number of cases (21-31%) were reported from 16-45 yr age group in both the genders while lower numbers (5-6.4%) were seen from elderly people above sixty years (*P*<0.05).

**Table I:** Distribution of Chikungunya outbreaks in different districts of Orissa

District	Block (No. of Village/Town)	Period of outbreak	Population at risk	No. of clinical cases	Case attack rate (%)

Sundergarh	Panposh town (1)	27.02.06 - 6.3.06	10233	5194	50.76
Gajapati	Parlakhemundi (1)	22.07.06 - 30.07.06	354	39	11.02
Ganjam	Keluapali (1)	21.08.06 - 30.08.06	401	70	17.46
Kendrapada	Mahakalapada (2)	08.09.06 - 25.10.06	2495	752	30.14
Cuttack	Mahanga (4)	13.09.06 - 14.10.06	1832	537	29.31
Nayagarh	Odagaon (4)	2.05.07 - 7.06.07	8820	932	10.57
Bhadrak	Barpada (1)	7.06.07 - 15.06.07	300	88	29.33
Ganjam	Beguniapada (1) Buguda (1) Polasara (3)	21.07.07 - 17.09.07	3000	50	1.67
Nayagarh	Bhapur (1) Khandapada (3)	20.08.07 - 25.09.07	1682	180	10.70
Cuttack	Niali (1)	27.08.07 - 4.10.07	500	2	0.40
Jajpur	Madhuban (1) Badachana (1) Bari (1) Rasulpur (4)	29.08.07 - 2.10.07	4890	462	9.45
Bhadrak	Dhamnagar (1)	1.09.07 - 12.09.07	3500	226	6.46
Kendrapada	Mahakalapada (7) Garadpur (10) Rajkanika Deabish (1) (3) Kendrapada (4)	1.09.07 - 6.11.07	26117	655	2.51
Puri	Nimapara (3) Satyabadi (1) Puri sadar (4) Gop (1) Astaranga (1)	4.09.07 - 3.11.07	4691	826	17.61
Jagatsinghpur	Nuagaon (3) Mandasahi (3)	20.09.07 - 10.10.07	6451	684	10.60
Khurda	Bankoi (2)	22.09.07 - 31.10.07	1024	80	7.81
Balesore	Bhogarai (2)	8.10.07 - 5.11.07	776	50	6.44
Keonjhar	Ananadpur (1)	10.10.07 - 3.11.07	800	40	5.00
Total district - 13	33 blocks (78)	Feb 06 - Nov 07	77866	10867	13.96

**Table II:** Age and sex distribution of CHIK cases in the State (2006-2007)

Age group (yr)	Number of Chikungunya cases		
	Male (%)	Female (%)	Total (%)

0-15	1111 (22.5)	1246 (20.9)	2357 (21.6)
16-30	1348 (27.3)	1876 (31.5)	3224 (29.6)
31-45	1255 (25.4)	1257 (21.1)	2512 (23.1)
46-60	18 (896.1)	1255 (21.1)	2151 (19.7)
>60	316 (6.4)	307 (5.1)	623 (5.7)
Total	4926 (45.3)	5941 (54.6)	10867

Statistical significance not shown; X^2^ *P*< 0.05 – as given in Results

**Fig:**
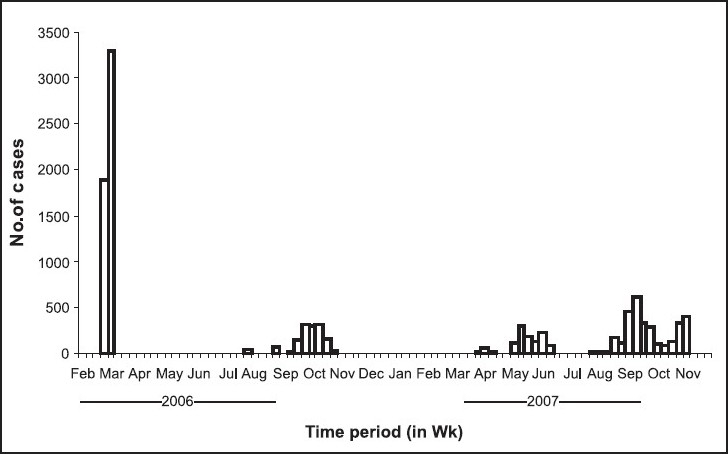
Epidemic curve showing week-wise onset of CHIK cases in Orissa (2006-2007).

*Signs and symptoms:* Acute onset of fever or joint pain was the initial symptom to present with. Common symptoms complained were fatigue (89.2%) and fever (87%). Fever was also associated with chills (37%), headache (63%) and head reeling (68%). Joint pain with/without swelling was present in 68.5 per cent individuals and it was with fever in 64 per cent cases. Joint pain started on first or second day of fever and involved both small and big joints of extremity and spine. Maculopapular rash often with itching was seen in both trunk and extremities in 35 per cent cases and haemorrhagic manifestations (nasal and gum bleeding) were observed in a few individuals (0.9%). Anorexia, nausea and pain abdomen were the other associated minor symptoms. Majority of the symptoms subsided within 3-5 days whereas joint pain and swelling persisted for more than 7 days. All the individuals were successfully treated symptomatically with common analgesics and antihistaminics. No death was observed due to the above illness. Average work days lost (expressed as numbers of days an individual was unable to perform his usual activity and remained confined to home) was up to 4 days(ranging from 2 - 7 days) in a majority of individuals.

*Laboratory confirmation:*Blood samples were collected from 725 individuals who volunteered for the investigation from different age and sex groups in the studied districts (Table III). The results confirmed that the outbreaks were due to CHIK virus infection. Seropositivity of the tested samples for CHIK IgM ranged from 3 to 44 per cent in the different outbreaks (Table IV). Dengue IgM test revealed positivity in a few samples from Nayagarh (n=3; 2.8%) and Puri (n=1; 0.8%) districts. Thick blood smears were examined for malaria parasiteand none of the blood smears were positive.

*Mosquito vector in the outbreak areas:*Adult mosquito and larval survey were conducted in outbreak affected villages of Cuttack, Nayagarh and Kendrapara districts (Table V). The density (PMHD) of *Ae. aegypti, Ae. albopictus* and *Ae. vittatus* were 4.2, 7.6 and 1.28 in Kendrapara, 3.7, 6.2 and 0.8 in Cuttack and 2.3, 8.0 and 1.6 in Nayagarh districts respectively. Adult mosquitoes were found both in the living rooms and Cattle sheds. The house, container and Breteau indices calculated were 19, 18.9 and 76 in Kendrapara, 17.2, 16.9 and 73.6 in Cuttack and 21.9, 20.1 and 84.7 in Nayagarh respectively.

**Table III:** Investigation for CHIK antibody (IgM) on blood samples collected from the outbreaks

Age group (yr)	Male		Female		Total	
	Samples tested	Chik IgM positive N (%)	Samples tested	Chik IgM positive N (%)	Samples tested	Chik IgM positive N (%)

0-15	44	9 (20.4)	65	16 (24.6)	109	25 (23)
16-30	65	18 (27.7)	68	14 (20.5)	133	32 (24)
31-45	80	23 (28.7)	74	17 (23)	154	40 (26)
46-60	100	16 (16)	119	32 (26.8)	219	48 (22)
>60	60	14 (23.3)	50	13 (26)	110	27 (24.5)
Total	349	80 (23)	376	92 (24.4)	725	172 (23.7)

**Table IV:** Seropositivity for CHIK and dengue IgM in the tested samples from different districts

Name of the district	No. of samples tested	No. of Chik IgM positives (%)	No. of dengue IgM positives (%)

Bhadrak	26	4 (15.38)	0
Ganjam	16	7 (43.75)	0
Jajpur	44	5 (11.36)	0
Kendrapada	153	40 (26.14)	0
Nayagarh	105	44 (41.9)	3 (2.8)
Khurda	18	5 (27.77)	0
Balesore	12	2 (16.66)	0
Puri	116	41 (35.34)	1 (0.8)
Keonjhar	10	3 (30)	0
Cuttack	163	16 (9.81)	0
Jagatsinghpur	32	1 (3.12)	0
Sundergarh	20	2(10)	0
Gajapati	10	2(20)	0
Total districts (13)	725	172 (23.7)	4 (0.5)

**Table V:** Results of survey for *Aedes larvae*

Districts	No. of HH searched for *Aedes larvae*	No of HH +ve for *Aedes larvae*	No of containers searched	No of containers +ve for *Aedes larvae*

Kendrapara	121	23	488	92
Cuttack	87	15	377	64
Nayagarh	105	23	442	89

## Discussion

India experienced outbreaks due to CHIK infection in 2005 after a long gap^9^ whereas Orissa with no history of CHIK infection experienced its first epidemic in 2006. After emergence of CHIK virus infection in the State, it spread widely affecting mostly rural areas from about half of the administrative districts. These villages were from different physiographic divisions of the State like coastal plane (Eastern Orissa), Northern plateau and Eastern Ghat (southern Orissa) which included both plane and hilly areas and it occurred within a period of one year and nine months. Clinical attack rate was up to 50 per cent in the different outbreak sites, which was also similar to reports from other States of India showing attack rate up to 45 per cent^10^. The seropositivity rate would have been increased if paired sera could be tested from the individual cases. The observed seroprevalence for CHIK IgM antibody varied between 3 to 44 per cent in tested samples from different districts and the overall prevalence rate was 13.9 per cent. Similar seropositivity rates were also noted from other affected States of India^10^,^11^

Though the illness was non fatal in all the outbreak sites, morbidity was high with loss of work and population affected rate as high as 50 per cent in the major outbreaks.

Three species of *Aedes, i.e., aegypti, albopictus* and *vitattus* were identified in the affected areas which are the possible vectors for transmission. The house and Breateau indices were observed to be much higher than the recommended indices of 5 and 20 respectively, which might have favoured transmission of the virus within the population^12^. Earlier reports had also shown the presence of *Aedes* species in varying densities in other regions of the State^13^–^16^ which indicated potential for spread to other areas. The CHIK virus is also known to spread through transovarial transmission in the mosquitoes and possible sylvatic cycles^17^.All these indicate possibility of spread of the infection to other areas of the region.

Though no community study on seroprevalence of CHIK antibody is available from the State, a cross-sectional survey carried out in Kolkota 10 years ago indicated low level (4.37% antibody positivity) of herd immunity to CHIK infection^18^. Hence the reasons for re-emergence of CHIK in the Indian subcontinent though not precisely known, might be due to a variety of social, environmental, behavioural and biological changes^19^,^20^. Alternatively, it was expected that the lack of herd immunity probably led to its rapid spread^21^.

In conclusion, the present report confirmed the emergence of CHIK virus infection in Orissa, India, in 2006 and its spread in the s0 tate affecting a wider geographic zone in a shorter period which was facilitated by presence of the *Aedes* vector species. The present findings will help the health authorities and the community physicians to keep vigil over the problem and taking steps for early diagnosis of the illness and undertaking preventive measures to curtail morbidity and spread.
